# Development and validation of cuproptosis-related lncRNA signatures for prognosis prediction in colorectal cancer

**DOI:** 10.1186/s12920-023-01487-x

**Published:** 2023-03-22

**Authors:** Lin Pang, Qingqing Wang, Lingxiao Wang, Zhen Hu, Chong Yang, Yiqun Li, Zhenqi Wang, Yaoping Li

**Affiliations:** 1 Department of Colorectal and Anal Surgery, The Fifth Clinical Medical College of Shanxi Medical University, Taiyuan, 030012 China; 2 Department of Health Statistics, School of Public Health, Shanxi Medical University, Taiyuan, 030001 China

**Keywords:** Colorectal cancer, Cuproptosis, LncRNAs, Genes signature, Prognostic model

## Abstract

**Background:**

Cuproptosis, a novel form of programmed cell death, plays an essential role in various cancers. However, studies of the function of cuproptosis lncRNAs (CRLs) in colorectal cancer (CRC) remain limited. Thus, this study aims to identify the cuprotosis-related lncRNAs (CRLs) in CRC and to construct the potential prognostic CRLs signature model in CRC.

**Methods:**

First, we downloaded RNA-Seq data and clinical information of CRC patients from TCGA database and obtained the prognostic CRLs based on typical expression analysis of cuproptosis-related genes (CRGs) and univariate Cox regression. Then, we constructed a prognostic model using the Least Absolute Shrinkage and Selection Operator algorithm combined with multiple Cox regression methods (Lasso-Cox). Next, we generated Kaplan–Meier survival and receiver operating characteristic curves to estimate the performance of the prognostic model. In addition, we also analysed the relationships between risk signatures and immune infiltration, mutation, and drug sensitivity. Finally, we performed quantitative reverse transcription polymerase chain reaction (qRT -PCR) to verify the prognostic model.

**Result:**

Lasso-Cox analysis revealed that four CRLs, SNHG16, LENG8-AS1, LINC0225, and RPARP-AS1, were related to CRC prognosis. Receiver operating characteristic (ROC) and Kaplan–Meier analysis curves indicated that this model performs well in prognostic predictions of CRC patients. The DCA results also showed that the model included four gene signatures was better than the traditional model. In addition, GO and KEGG analyses revealed that DE-CRLs are enriched in critical signalling pathway, such as chemical carcinogenesis-DNA adducts and basal cell carcinoma. Immune infiltration analysis revealed significant differences in immune infiltration cells between the high-risk and low-risk groups. Furthermore, significant differences in somatic mutations were noted between the high-risk and low-risk groups. Finally, we also validated the expression of four CRLs in FHCs cell lines and CRC cell lines using qRT-PCR.

**Conclusion:**

The signature composed of SNHG16, LENG8-AS1, LINC0225, and RPARP-AS1, which has better performance in predicting colorectal cancer prognosis and are promising biomarkers for prognosis prediction of CRC.

**Supplementary Information:**

The online version contains supplementary material available at 10.1186/s12920-023-01487-x.

## Introduction

Colorectal cancer (CRC) is the third most common cancer, behind breast cancer and lung cancer. A total of 1.93 million colorectal cancer cases were diagnosed with 0.93 million deaths worldwide in 2020, ranking second among cancer deaths [1]. Although therapeutic measures of CRC are available, patient prognosis remains unsatisfactory due to tumour recurrence and metastasis [2, 3]. The number of CRC cases is expected to increase by 66% from 1.93 million in 2020 to 3.20 million in 2040 [4]. Thus, it is crucial to seek potential risk signatures to improve the prognosis of CRC.

Long non-coding RNAs (IncRNAs) are non-coding RNAs greater than 200 nucleotides in length that are unique genes with regulatory functions but are not transcribed [5]. The latest evidence suggests that many IncRNAs are associated with the occurrence and progression of cancer and are perceived as new therapeutic targets [6, 7]. The lncRNA SNHG16 directly regulates the miR-195/SREBP2 axis to enhance the progression of pancreatic cancer [8]. The p53RRA-G3BP1 interaction suppresses lung cancer progression via cell cycle arrest and ferroptosis [9]. Circ-0007142 is overexpressed in CRC and inhibits CRC cell proliferation by promoting apoptosis and ferroptosis [10]. The lncRNA SNHG16 is involved in the proliferation, migration, and epithelial-mesenchymal transition of CRC through the miR-124-3p/MCP-1 axis [11]. LINC00312 represses CRC cell proliferation and invasion by regulating miR-21 [12]. Research shows that 15-lncRNA can be used as a prognostic indicator for CRC to predict the survival of CRC patients [13].

In 2022, Tsvetkov and his colleagues proposed a new concept, "cuproptosis," which is copper dependent, regulated, and a novel form of cell death distinct from other known forms of programmed cell death [14]. Mechanisms of cuproptosis: Copper is directly bound to the lipoylated components of the tricarboxylic acid (TCA) cycle, leading to lipoylated protein aggregation and subsequent iron-sulfur cluster protein loss that could cause proteotoxic stress and ultimately cell death [15]. Studies have revealed the role of ferroptosis-related lncRNAs in several cancers, including breast cancer [16–18], lung cancer [19–21] and colorectal cancer [22–25]. However, studies of the function of cuproptosis lncRNAs (CRLs) in colorectal cancer (CRC) remain limited. We hypothesize that cuproptosis-related lncRNAs may also affect CRC cells based on previous studies [14–25].

In this study, we obtained RNA-sequencing profile data from TCGA and constructed a prognostic model containing four CRLs, and we verified the predictive accuracy of the model using internal and external cohorts. We found that risk signatures were not only an independent prognostic factor but also predicted the clinical status of CRC patients. We also conducted enrichment analysis to analyse carcinogenic pathways between different signatures. Moreover, we further analysed the mechanisms of CRLs in CRC using a series of methods, such as immune infiltration analysis, mutation analysis, and drug sensitivity analysis. Finally, we verified the expression of SNGH16, LINC02257, PRARP-AS1, and LENG8-AS1 in FHCs cell lines and CRC cell lines.

## Materials and methods

### Data collection and processing

RNA-sequencing profile data of colorectal cancer tumour samples, including 473 tumour samples and 41 normal samples, were downloaded from The Cancer Genome Atlas datasets (TCGA, https://tcga-data.nci.nih.gov/tcga/) and served as a training dataset. Data with incomplete follow-up times were excluded. Finally, a total of 446 samples were included in the present study. In addition, the RNA-sequencing profile data of external validation datasets, including GSE152430 (49 samples), GSE192667 (89 samples), and GSE190826 (117 samples), were obtained from Gene Expression Omnibus (GEO, https://www.ncbi.nlm.nih.gov/geo) datasets.

The probe-identified gene matrix files were transformed into gene symbols based on the annotation patterns obtained by the relevant platforms. We examined each clinical index and excluded patients with missing clinical information or lacking complete follow-up information. Then, batch effects of three datasets of GEO were removed by the “sva” R package and were merged. Finally, we extracted the lncRNA expression data of TCGA and GEO, respectively, extracting the intersection of lncRNA expression data between the TCGA and GEO.

### Identification of cuproptosis-related LncRNAs

First, we obtained ten cuproptosis-related genes (FDX1, LIAS, LIPT1, DLD, DLAT, PDHA1, PDHB, MTF1, GLS, and CDKN2A) based on the studies by Tsvetkov (https://doi.org/10.1126/science.abf0529) [14]. Then, gene expression data from CRC cancer patients in TCGA cohort were subdivided into mRNA and lncRNA according to the gene type. Finally, we used Spearman’s correlational analysis to identify the effects of cuproptosis-related lncRNAs on the expression levels of the cuproptosis-related genes. Cut-off criteria of the Spearman’s correlation coefficient > 0.4 and *P* value < 0.001 were employed to identify genes significantly related to CRC prognosis. In this study, the IncRNAs related to cuproptosis were regarded as cuproptosis-related lncRNAs (CRLs). The correlation analyses were performed by the R package “limma”.

### Construction of the CRLs risk signatures

First, TCGA datasets were randomly split into training (50%) and internal validation (50%) cohorts. Then, the relationship between CRLs and CRC patient prognosis was analysed by univariate Cox regression (*P* < 0.05). Moreover, the results of univariate Cox regression were analysed based on the least absolute shrinkage and selection operator (LASSO) to prevent the overfitting of data. Next, multivariate Cox regression was established to analyse the results of the LASSO model. The following risk score formula was employed: $\sum {\beta_{{\text{i}}} } \times {\text{EXP}}\left( {\textsl{IncRNA}} \right)_{{\text{i}}}$, where *β*_i_ is the regression coefficient and $\textsl{EXP} \left( {{\text{Inc}}\;\textsl{RNA}} \right)$ is the expression of each lncRNA. Training datasets were sorted into high-risk and low-risk groups based on the median risk score. Moreover, we calculated the risk score of each CRC patient in the internal testing set (TCGA) and external validation set (GEO cohort) based on the same risk score system used for the training set and divided the internal testing set and external validation set into high-risk and low-risk groups based on the median risk score. Besides, we verified the distribution of risk CRLs in the high-risk and low-risk groups using the principal component analysis method (PCA).

### Correlation between the risk score and clinical characteristics

Wilcoxon and Kruskal–Wallis tests were used to explore the correlation between risk score and clinical characteristics, including age, sex, AJCC stage, and T, N and M stage.

### Survival analysis of the risk scores

To assess the predictive value of CRLs in the prognosis of CRC, we analysed the survival difference between the high-risk and low-risk groups using the Kaplan–Meier method and log-rank test. Furthermore, the area under the receiver operating characteristic (ROC) curve was utilized to evaluate the predictive performance of CRLs.

### External validation of the risk score

We verified the predictive value of CRLs in the GEO cohort to elucidate its predictive ability. Then, we calculated the risk score of each CRC patient in the GEO cohort based on the same risk score of TCGA training set and analysed the survival difference between the high-risk and low-risk groups using the Kaplan–Meier method and log-rank test. In addition, the area under the receiver operating characteristic curve was calculated to illustrate the predictive effect of the risk signature.

### Prognosis of risk score and clinical characteristics

Stratification analysis of clinical characteristics, including age, sex, stage, and T, N, M stage, was employed to further explore the predictive value of risk score in clinical conditions. The log-rank test and univariate Cox analysis were performed to determine the survival status of the high-risk and low-risk groups.

### Construction of the nomogram

A nomogram combining the clinical characteristics and risk score was created to help clinicians to predict the survival of CRC patients. Clinical characteristics and risk scores were included in the construction of the nomogram model based on multivariate Cox regression analysis. The calibration curve was used to evaluate the prediction accuracy between the actual and predicted survival. We also compared the predictive performance of the nomogram model with other clinical characteristics using the area under the ROC curve. In addition, we generated a decision clinical analysis (DCA) curve to assess the net benefit of risk scores in clinical conditions.

### Enrichment analysis

To reveal the potential function of CRL-related risk signatures, Gene Ontology (GO) and Kyoto Encyclopedia of Genes and Genomes (KEGG) were used to identify the potential functional pathways using the "clusterProfiler" and "enrichplot" packages in R. The *P* value < 0.05 was considered statistically significant.

### Assessment of the correlation between tumour-infiltrating immune cells and risk score

We used the deconvolution algorithm [26] to calculate the abundance of tumour-infiltrating immune cells (TIICs) in each CRC patient in TCGA cohort to explore the correlation between the risk score and TIIC characteristics. In addition, we analysed the difference in the immune cell abundances in high-risk and low-risk groups using the Wilcoxon test, and results are presented as box plots.

### Gene mutations in the high-risk and low-risk groups

To compare the difference between mutant genes in the high-risk and low-risk groups, we applied "maftools" (R packages) to analyse and visualize the mutation profiles (MAF) data. In addition, we calculated the tumour mutation burden (TMB) of each CRC patient in TGCA datasets and compared the difference in TMB between the high-risk and low-risk groups based on T test. Moreover, we divided the CRC patients into high-mutation and low-mutation groups based on the median TMB and further generated survival curves to assess the performance of the risk score in the survival prediction of TMB.

### Drug sensitivity analysis

To identify whether the risk signature was related to CRC resistance, the "pRRophetic" [27] (R package) was used to predict the IC50 of the chemotherapeutic drug. The difference between groups was assessed by the Wilcoxon signed-rank test.

### Cell culture

Human intestinal epithelial cells (FHCs) and human colorectal cancer cell lines (SW480, SW620, HCT8, HT29, LoVo) were purchased from Procell Life Science&Technology Co. LTD. All cells were cultured in F-12 K, Leibovitz’s L-15 medium, RPMI 1620 medium (Hyclone, United States) containing 10% fetal bovine serum (Gibco BRL, United States) and 5% Pen-Strep solution (Bilolgical, Industries, China) at 37 °C, 95% humidity, and a 5% CO_2_ cell incubator.

### RNA extraction and real-time quantitative PCR (RT-qPCR) analysis

Total RNA was extracted using Trizol reagent (Takara, Japan) and reverse transcribed to cDNA using the PrimeScript RT Master Mix (Takara, Japan). The RT-qPCR analyses were performed in triplicate using the NovoStart SYBR qPCR SuperMix Plus Kit (Novoprotein, China) and detected using an Applied Biosystems 7500 Real-Time PCR System (Thermo Fisher Scientific, USA). β-actin was chosen as an internal reference. The comparative Ct approach was used to calculate the fold-changes in relative gene expression (fold change = 2^−△△Ct^). The primers used in real-time PCR were listed in Additional file 1: Table S1 and purchased from Sangon Biotech (Shanghai, China).

### Detection of expression of SNHG16, LINC02257, RPARP-AS1, LENG8-AS1 in colorectal cancer cell by RT-qPCR

Of 6 Colorectal cancer cell tissues were reversed transcribed to cDNA that were β-actin calibrated were purchased Shanghai Outdo Biotech (Shanghai, China). RT-qPCR was used to examine the expression of SNHG16, LINC02257, RPARP-AS1, LENG8-AS1 using an Applied Biosystems 7500 Real-Time PCR System (Thermo Fisher Scientific, USA).

### Statistical analysis

Spearman correlation analysis was used to explore the correlation between cuproptosis-related genes (CRGs) and CRLs. The chi-square test was applied to analyse differences in the proportions of clinical characteristics in the training set and testing set. Student's t test was used to compare the TMB in the high-risk and low-risk groups, and the Wilcoxon test was applied to identify the IC50 in the high-risk and low-risk groups. All statistical analyses were performed using R software and its appropriate packages, and the *P* value < 0.05 was considered statistically significant.

## Results

### Identification of cuproptosis-related LncRNAs in CRC

**Fig. 1:**
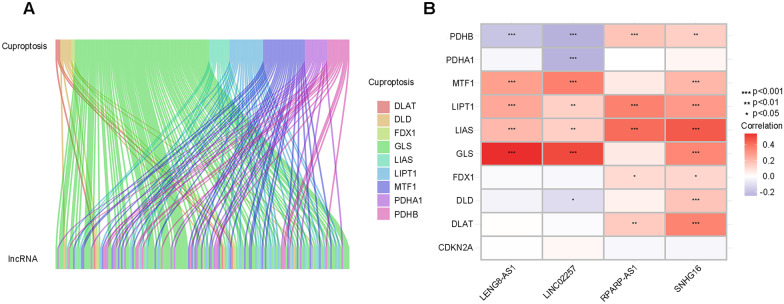
Sankey diagram showing the detailed connection between cuproptosis-related genes and cuproptosis-related lncRNAs (**A**). The correlation between 9 cuproptosis-related genes and 4 prognostic cuproptosis-related lncRNAs, *p < 0.05, **p < 0.01 and ***p < 0.001 (**B**)

The study design was shown in Additional file 2: Fig. S1. A total of 424 cuproptosis-related lnRNAs (CRLs) were identified based on the set cut-off criteria ($\left| {{\text{R}}^{2} } \right| > 0.4$ and *P* < 0.001). A Sankey diagram was generated to show the connection between CRGs and CRLs (Fig. 1A). A heatmap was plotted to show the correlation between 10 CRGs and 4 CRLs (SNHG16, LENG8-AS1, LINC02257 and RPARP-AS1) (Fig. 1B).

### Construction of the CRL Risk signatures model

**Fig. 2:**
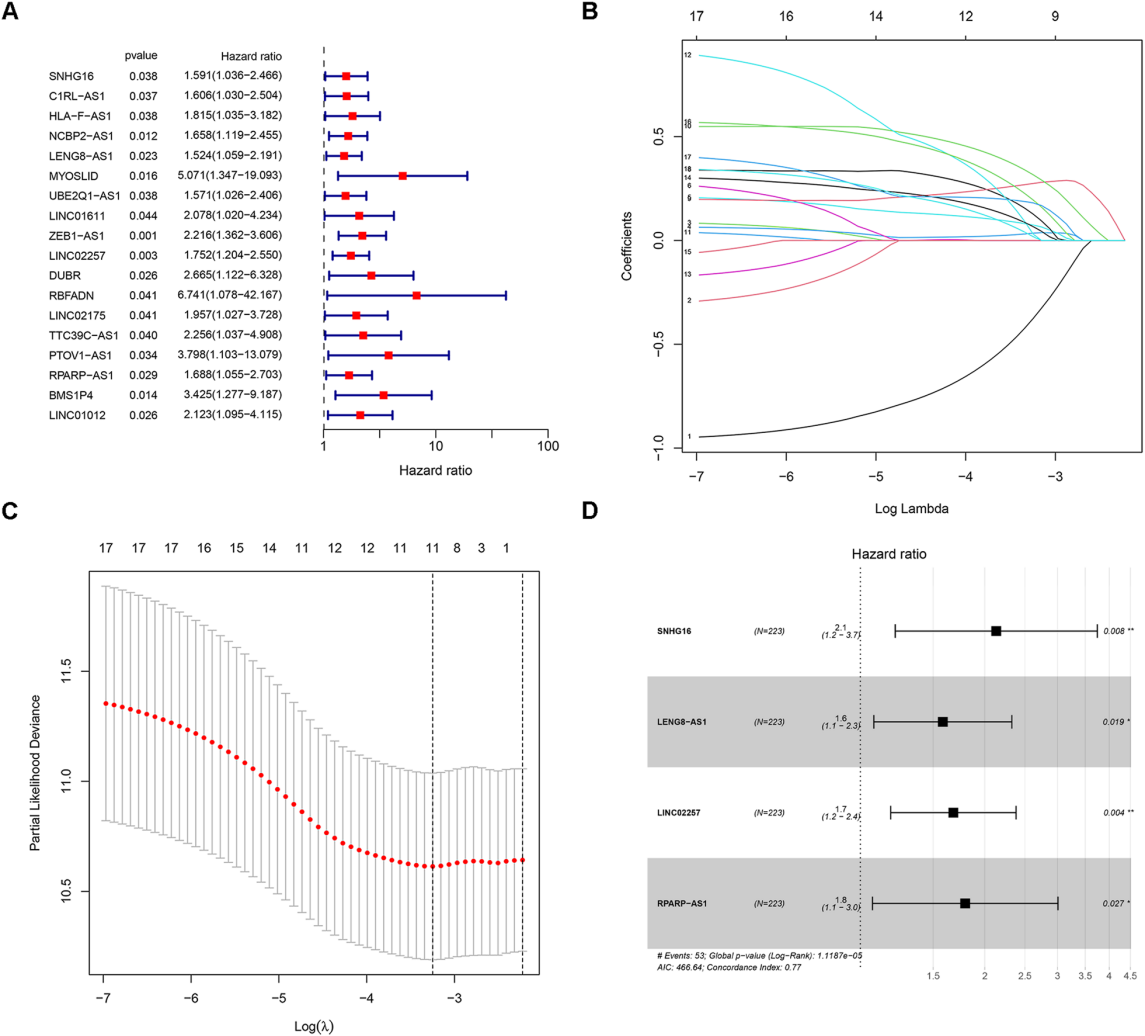
Univariate Cox analysis for the expression of cuproptosis-related lncRNAs (**A**). LASSO coefficient profiles of the cuproptosis-related lncRNAs (**B**). Partial likelihood deviance of different numbers of variables calculated via the LASSO regression model. LASSO coefficients of four cuproptosis-related lncRNAs in CRC (**C**). Multivariate Cox analysis for the expression of cuproptosis-related lncRNAs (**D**)

TCGA datasets were randomly split into training (223 samples) and internal validation (223 samples) set, and clinical information of the training and internal validation set were shown in Additional file 3: Table S2. First, we identified 18 CRLs associated with the prognosis of CRC via univariate Cox analysis (Fig. 2A) (*P* < 0.05). Then, we constructed predictive models using 18 CRLs and plotted their AUC (Additional file 2: Fig. S2). Second, 4 CRLs associated with the prognosis of CRC were selected by Lasso regression analysis with tenfold cross-validation (Fig. 2B, C). Besides, we have compared the model containing 18 CRLs to the model combined 4 CRLs. We found that the 18-CRLs model's performance was lower, as seen by AUCs of 1-, 3, and 5-year survival rates were 0.609, 0.598, and 0.534, respectively (Additional file 2: Fig. S3). Finally, four CRLs were identified as independent prognostic factors (Fig. 2D). The risk score of each CRC patient was calculated based on the Cox regression coefficient and the expression of 4 CRLs. Risk score = (0.75711 × EXP_SNHG16_) + (0.45962 × EXP_LENG8-AS1_) + (0.51846 × EXP_LINC02257_) + (0.58430 × EXP_RPARP-AS1_).

**Fig. 3:**
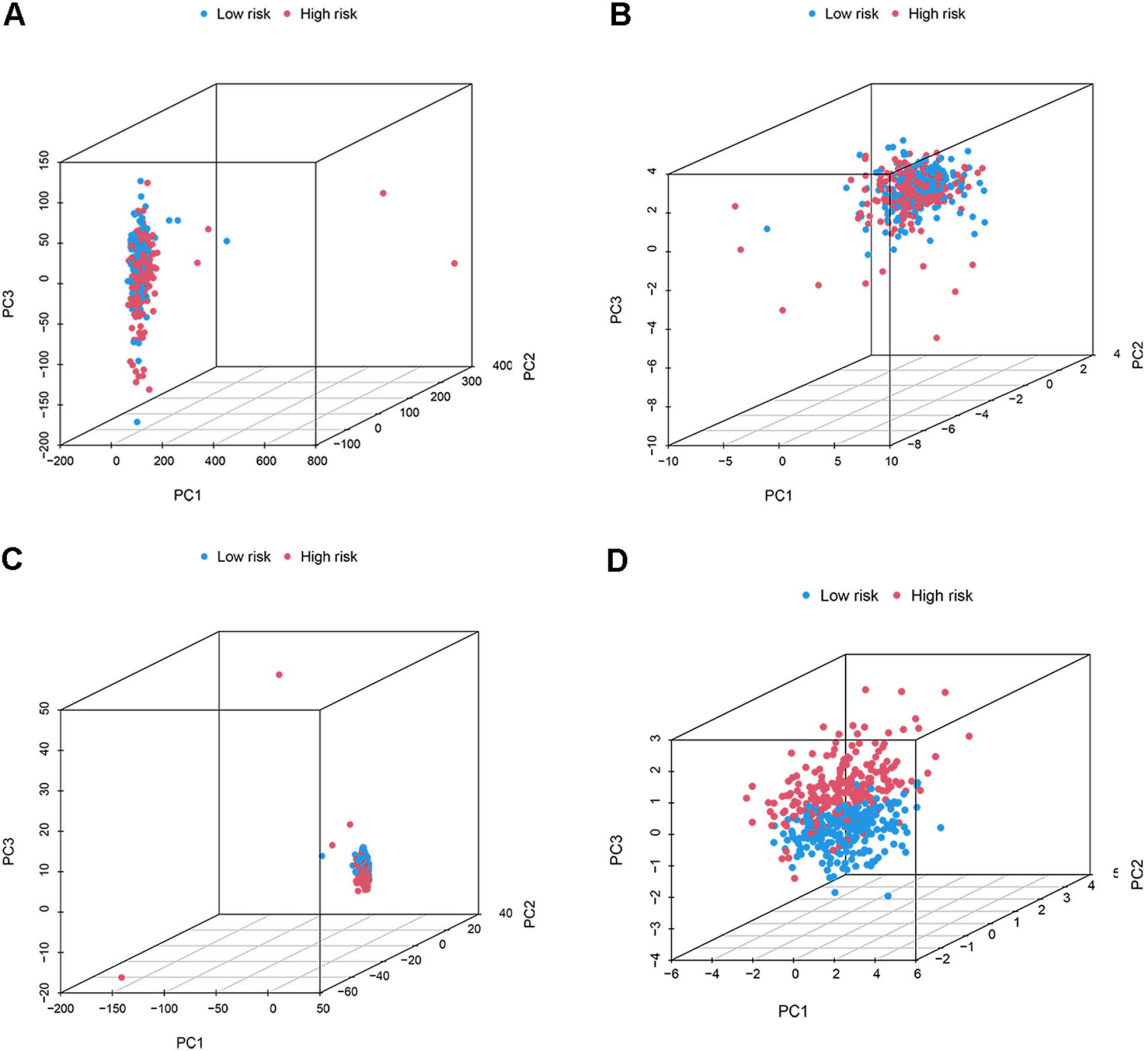
3D-PCA plots of all genes (**A**), cuproptosis genes (**B**), cuproptosis lncRNAs (**C**) and risk lncRNAs (**D**)

In addition, PCA indicated that the 4 risk-associated CRLs were able to differentiate CRC patients from TCGA cohort into different risk levels (Fig. 3).

### Correlation between the risk score and clinical characteristics

**Fig. 4:**
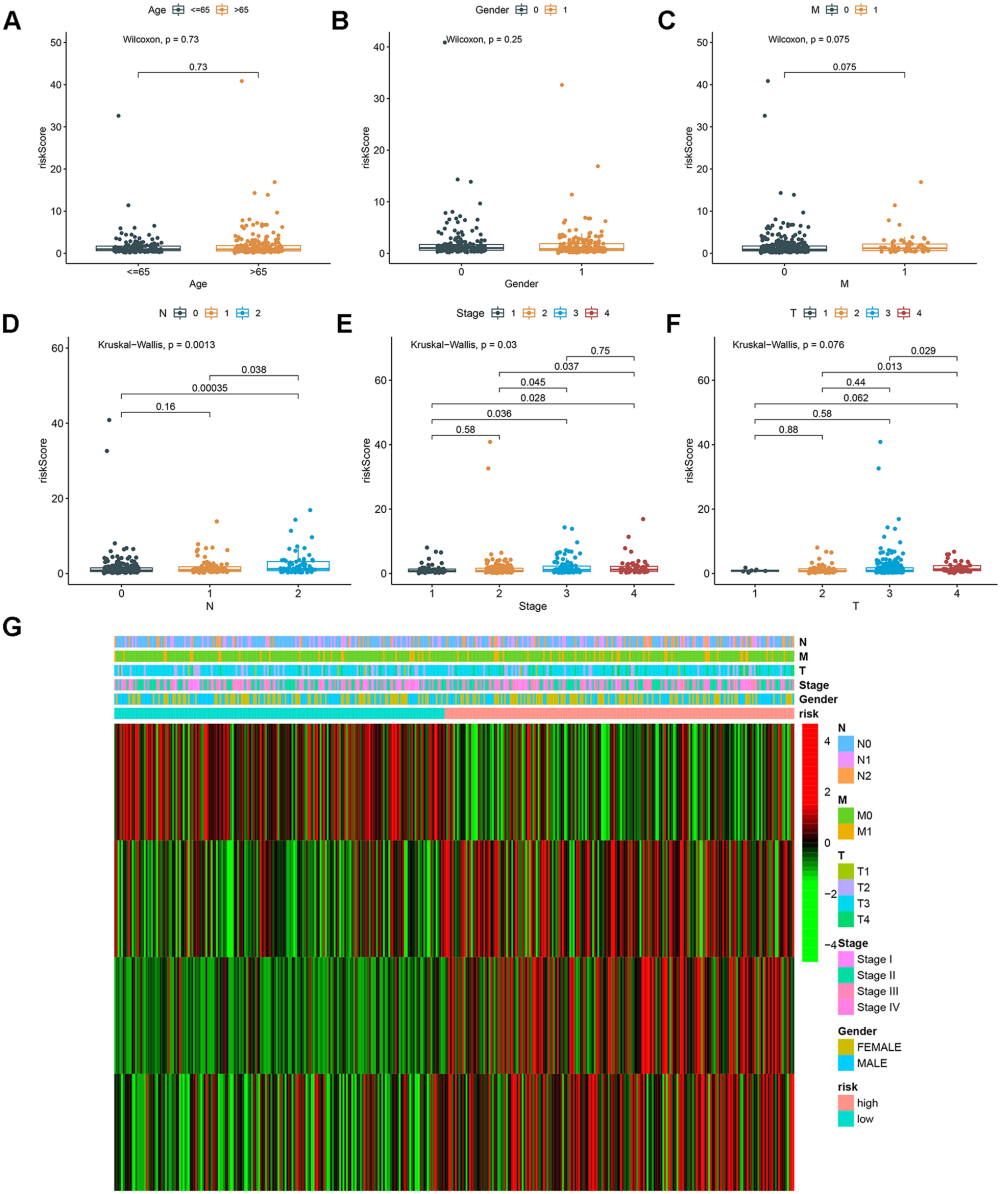
Correlation analysis between risk score and age (**A**), gender (**B**), M stage (**C**), N stage (**D**), AJCC stage (**E**), and T stage (**F**). Expression profiles of CRLs in each patient with CRC (**G**)

We explored the relationship between the risk score and clinical characteristics using the Wilcoxon and Kruskal–Wallis tests. Figure 4 shows significant correlations between the risk score and clinical characteristics (age, gender, AJCC stage, T stage, N stage and M stage).

### Survival analysis of risk score

**Fig. 5:**
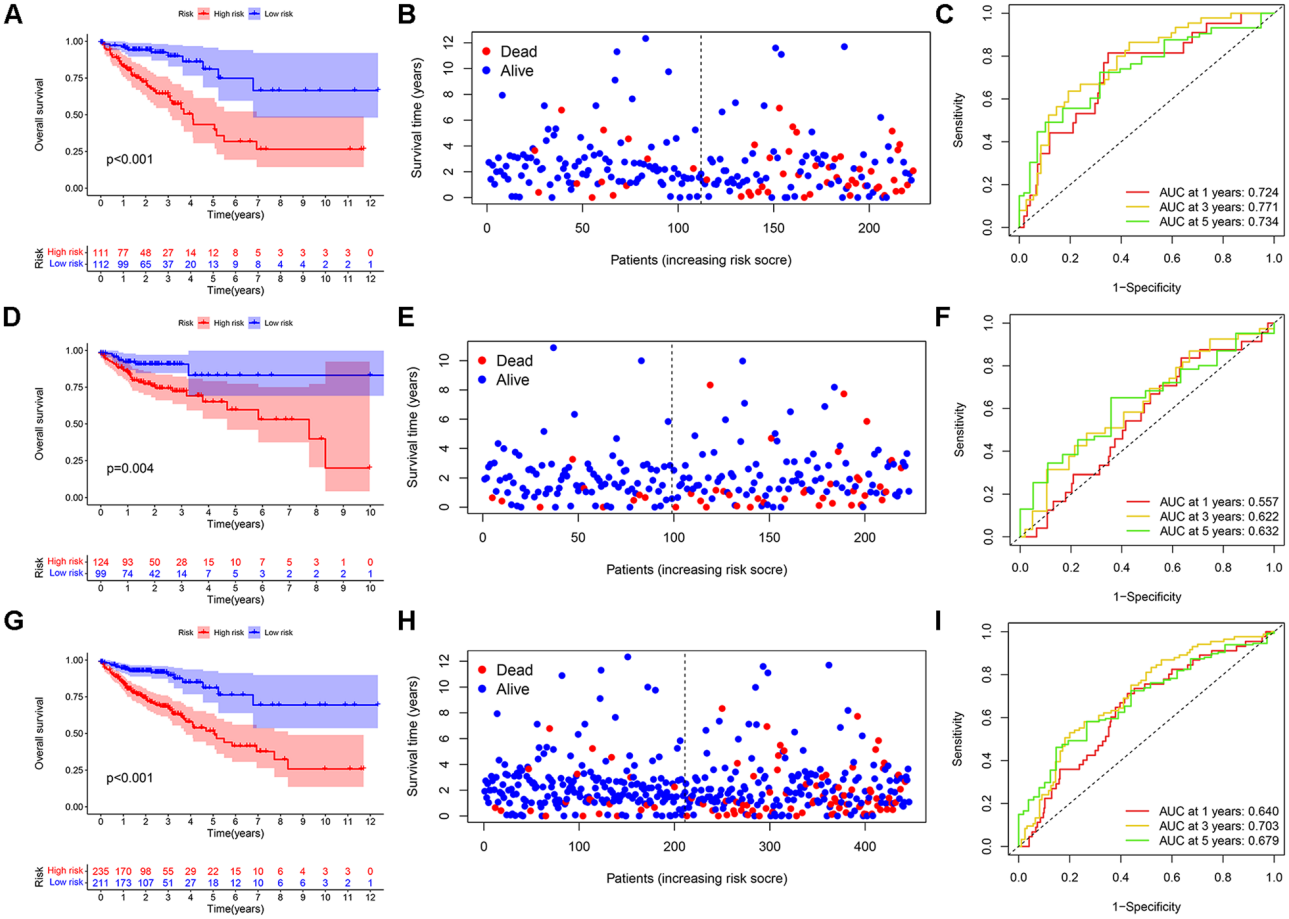
Kaplan–Meier survival plot for overall survival in TCGA training set (**A**), TCGA testing set (**D**) and total cohorts (**G**). Risk score plots for overall survival in TCGA training set (**B**), TCGA testing set (**E**) and total cohorts (**H**). Area under the receiver operating characteristic curve for the risk score of CRLM-based prognostic features at 1, 3 and 5 years in the TCGA training set (**C**), TCGA testing set (**F**) and total cohorts (**I**)

The testing set, total datasets, and GEO cohort were sorted into high-risk and low-risk groups based on the median risk score in the training set (0.991). Kaplan–Meier analysis and the log-rank test showed that the prognosis of patients in the high-risk group was worse than that of patients in the low-risk group (Fig. 5A). The risk plots revealed that the mortality rate of CRC patients in the high-risk group was more significant than that in the low-risk group and increased with the risk score (Fig. 5B). Furthermore, the ROC curve showed that the AUCs of 1-, 3- and 5-year survival in TCGA training cohort were 0.724, 0.771, and 0.734, respectively (Fig. 5C). The survival curves showed that CRC patients in the low-risk TCGA testing set had a higher probability of survival (Fig. 5D). In addition, the mortality rate of CRC patients in the TCGA testing set increased with the risk score (Fig. 5E). The AUCs of the ROC were 0.557, 0.622, and 0.632 for 1-, 3- and 5-year survival in the testing cohort, respectively (Fig. 5F). The prognosis of CRC patients in the high-risk group was worse than that in the low-risk group in all datasets (Fig. 5G). The risk plots show that CRC patients' mortality rate in total datasets increased with risk score (Fig. 5H). The AUCs of the ROC curves for 1-, 3, and 5-year survival rates based on total datasets were 0.640, 0.703, and 0.679, respectively (Fig. 5I).

### External validation of the cuproptosis-related LncRNA signatures model

**Fig. 6:**
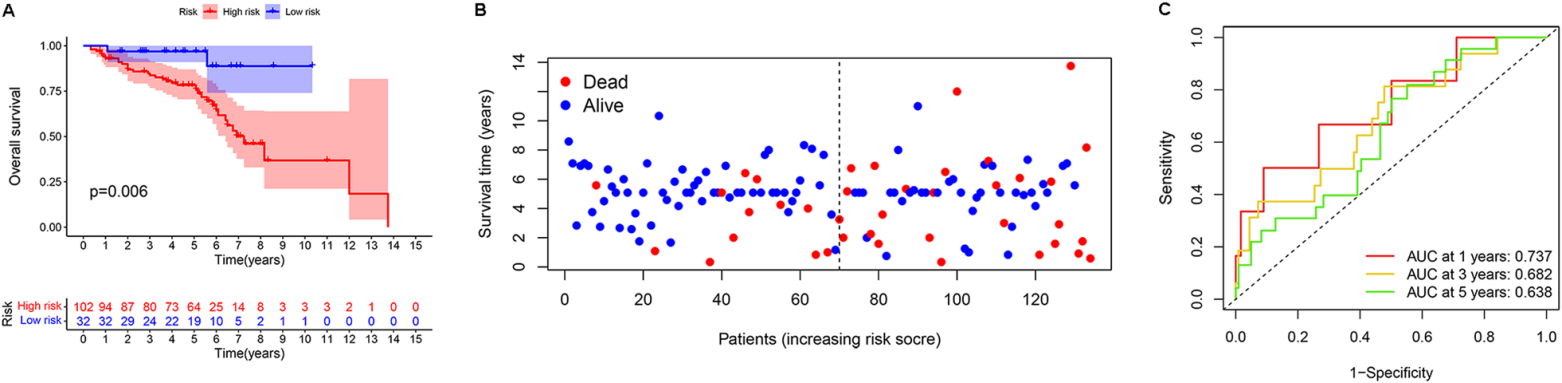
Kaplan–Meier survival plot and risk plot for overall survival in GEO cohorts (**A**, **B**). Area under the ROC curve for the risk score of CRLM-based prognostic features at 1, 3 and 5 years in GEO cohorts (**C**)

In addition, we further verified the prognostic value of CRLs based on the GEO cohort. The survival curves showed that CRC patients in the high-risk GEO cohort had worse survival outcomes (Fig. 6A). The risk plots revealed that the mortality rate increased with the risk score in the GEO cohort (Fig. 6B). Correspondingly, the 1-, 3-, and 5-year AUCs for predicting prognosis were 0.737, 0.682, and 0.638, respectively (Fig. 6C).

### Prognosis analysis between risk score and clinical characteristics

**Fig. 7:**
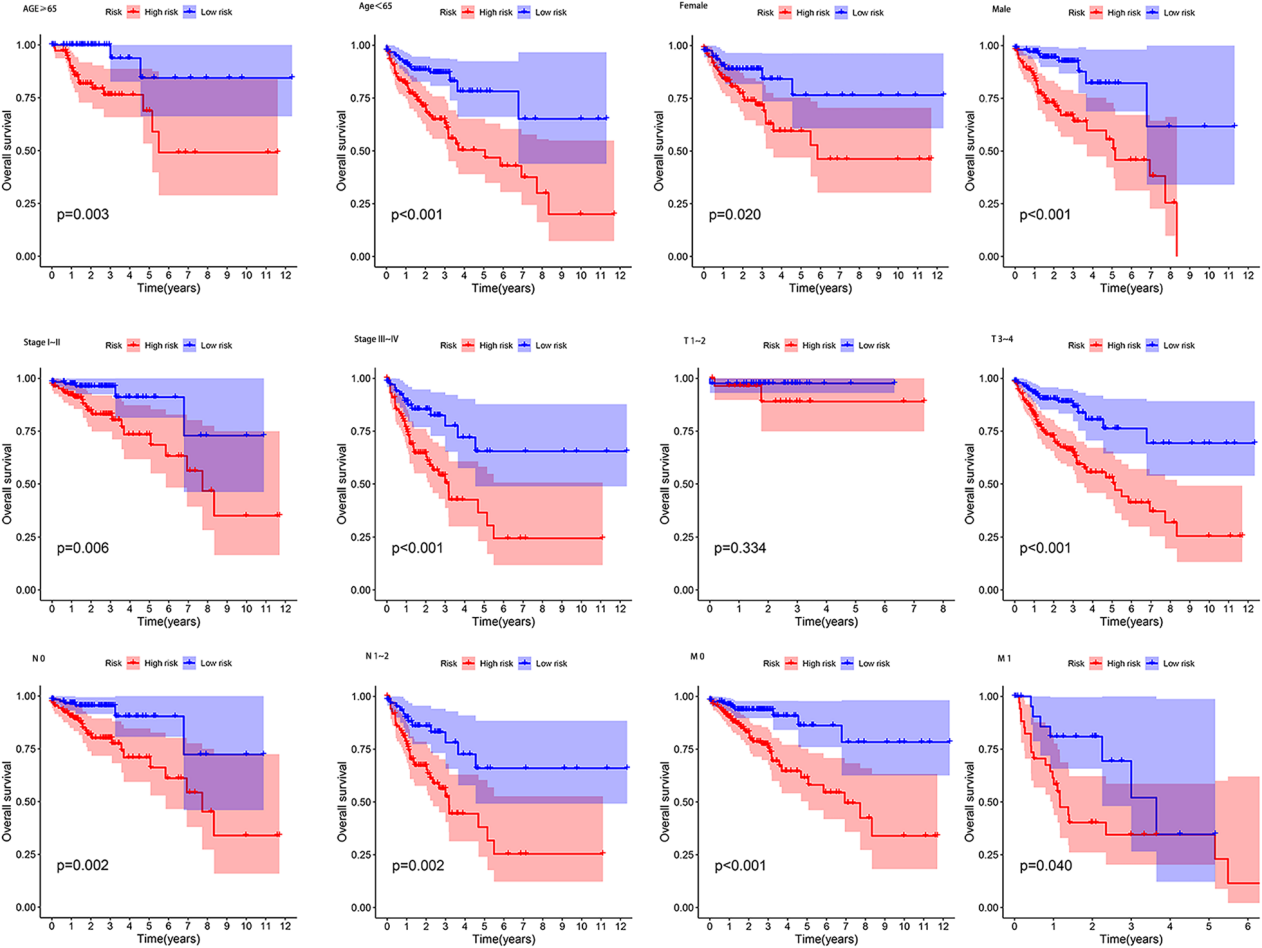
Kaplan–Meier survival plots of the 4 cuproptosis-related genes in TCGA datasets with different clinical characteristics (age, gender, stage I-II, stage III-IV, T1-T2, T3-T4, N0, N1-N2, M0, and M1)

We performed stratification analysis to explore further the predictive value of risk scores in clinical conditions. The results showed that the differences in the clinical characteristic subgroups between the high-risk and low-risk groups were statistically significant (*P* < 0.05), and the prognosis of the low-risk group was better than that of the high-risk group (Fig. 7). These findings indicate that CRLs play a vital role in the prognostic prediction of clinical conditions.

### Independent prognostic analysis

**Fig. 8:**
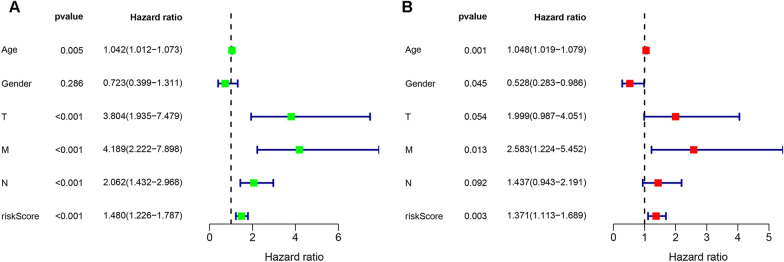
Forest plots of the univariate (**A**) and multivariate (**B**) Cox regression analyses of risk score and clinical features regarding prognostic value

To determine whether risk scores were independent risk factors for the survival of CRC patients, we constructed univariate and multiple Cox models to assess the relationship between risk scores and the survival of CRC patients. As shown in Fig. 8A, B there are significant relationships between risk score and the survival of CRC patients (*P* < 0.001, *P* = 0.003).

### Construction of the prognostic nomogram

**Fig. 9:**
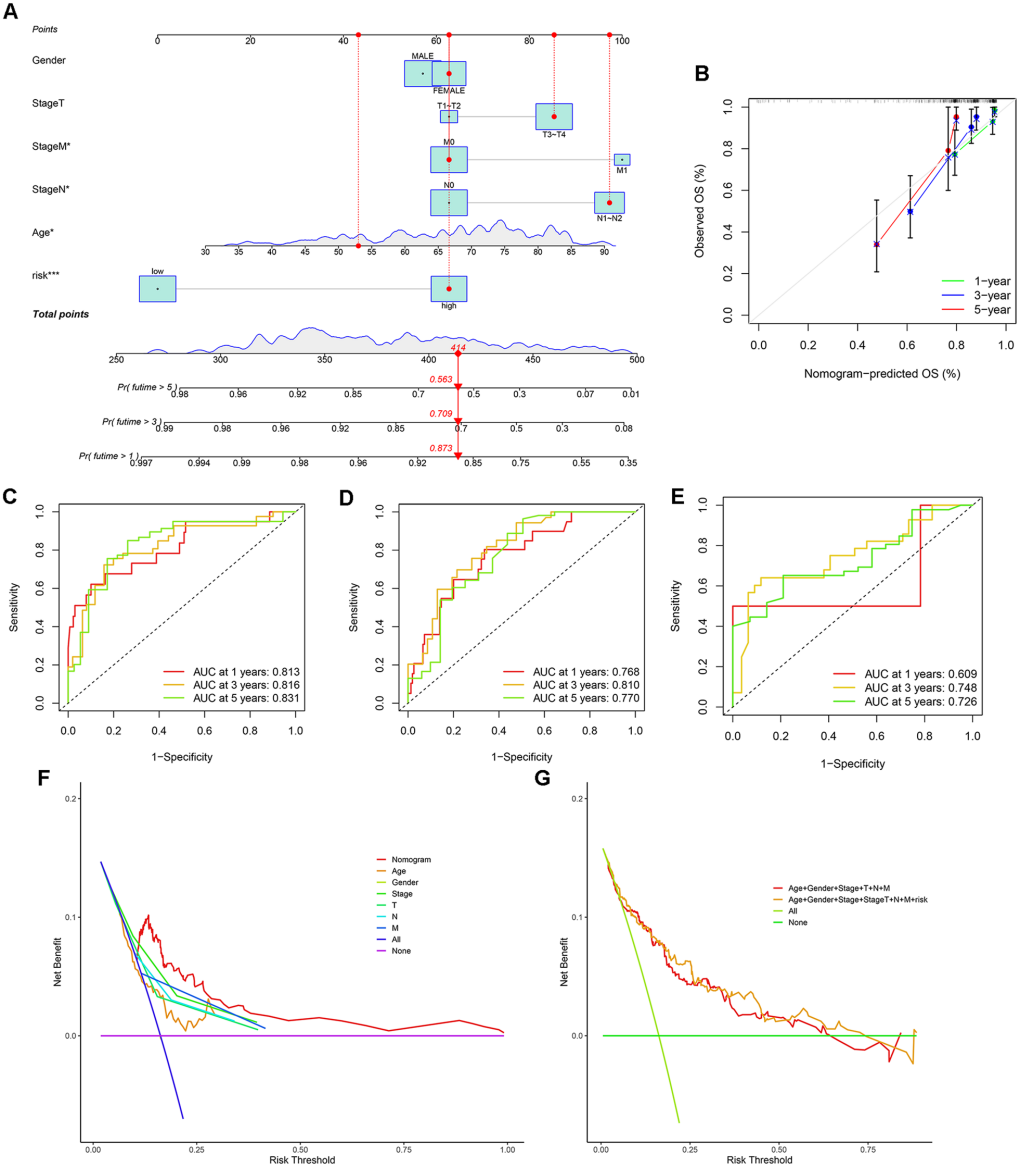
Nomogram incorporating the risk score and clinical characteristics to predict the 1-, 3- and 5-year overall survival rates of patients with CRC (**A**). The calibration curve for evaluating the nomogram Model (**B**). The area under the ROC curve incorporating the risk score and clinical characteristics to predict the 1-, 3- and 5-year overall survival rates of patients with CRC on TCGA training set (**C**), TCGA testing set (**D**) and GEO cohorts (**E**). Decision curve analysis of the nomogram (**F**), risk score and clinical characteristics (**G**)

We constructed a nomogram model integrating clinical characteristics and the risk score to predict the survival rate of CRC patients at 1, 3, and 5 years (Fig. 9A). The calibration plot revealed that the nomogram model exhibited excellent performance (Fig. 9B). Besides, we used the function “sbrier” to calculate the brier score, the score of 1-, 3- and 5-years are 0.245, 0.267 and 0.269, respectively. The ROC curves of 1-, 3- and 5-year survival rates based on TCGA training set were 0.813, 0.816 and 0.831, respectively (Fig. 9C). The ROC curves of 1-, 3- and 5-year survival rates based on TCGA testing set were 0.768, 0.810 and 0.770, respectively (Fig. 9D). The ROC curves of 1-, 3- and 5-year survival rates based on GEO cohorts set were 0.609, 0.748 and 0.726, respectively (Fig. 9E). In addition, decision clinical analysis (DCA) was applied to evaluate the net benefit in clinical conditions of the nomogram. The DCA curve showed that the net benefits of the nomogram model were better than those of other clinical characteristics, and the model containing the risk score and clinical characteristics was also better than the traditional model combined with clinical characteristics (Fig. 9F, G). Besides, we plotted the ROC curves to assess the performance of the nomogram model and clinical indicators. The results showed that the AUC values of the nomogram model were better than the model just with clinical indicators (Additional file 2: Fig. S4).

### Functional enrichment analysis

**Fig. 10:**
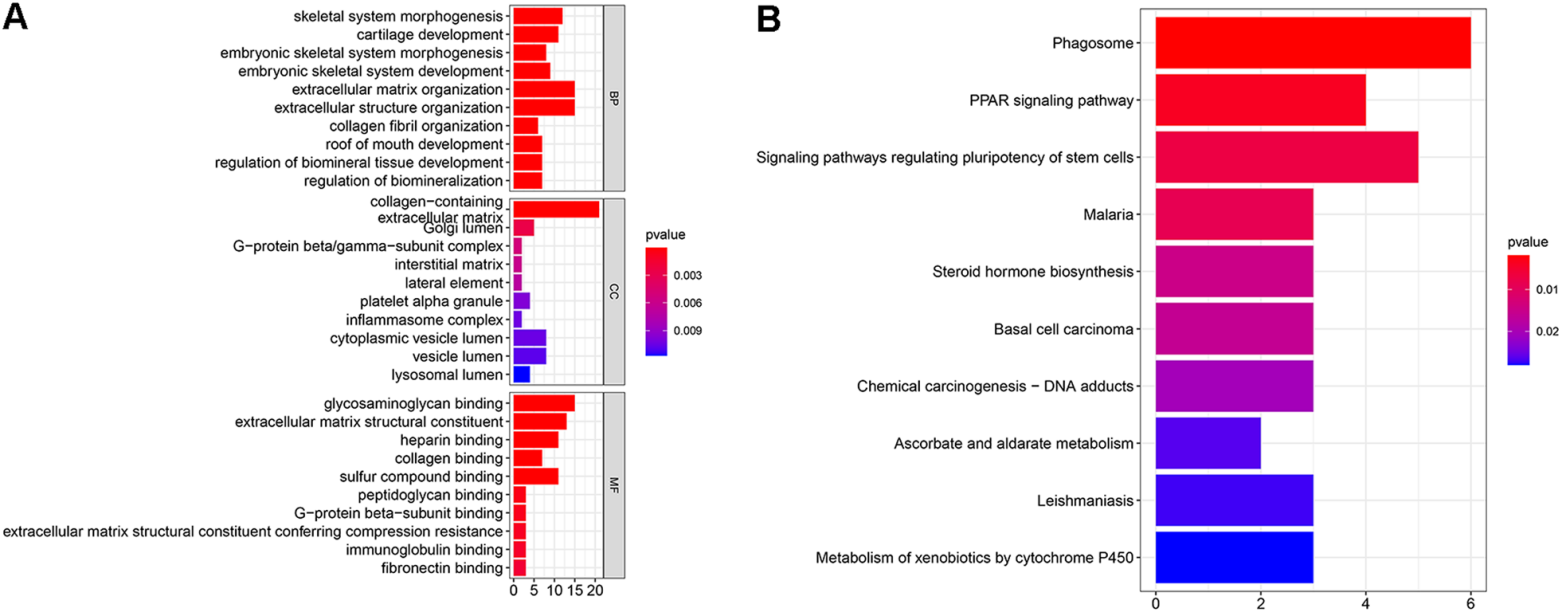
The top 10 biological processes, cellular components, molecular functions (**A**) and KEGG (**B**) pathways are illustrated

To reveal the biological function of CRLs, GO and KEGG analyses were performed on differentially expressed CRLs (DE-CRLs) between the high-risk and low-risk groups. GO functional analysis showed that the DE-CRLs were mainly involved in biological process (BP), molecular function (MF), and cell component (CC). Biological Process: extracellular structure organization, extracellular matrix organization and regulation of biomineral tissue development. Molecular Function: fibronectin binding. Cell Component: inflammasome complex. KEGG analysis revealed that the DE-CRLs were primarily enriched in the PPAR signalling pathway and signalling pathway regulating pluripotency of stem cells. Terms related to canceration included chemical carcinogenesis—DNA adducts and basal cell carcinoma. In addition, metabolism of xenobiotics by cytochrome P450 and steroid hormone biosynthesis were also implicated. The top 10 enriched terms are shown in (Fig. 10A, B).

### Estimation of tumour-infiltrating immune cells and association with risk score

**Fig. 11:**
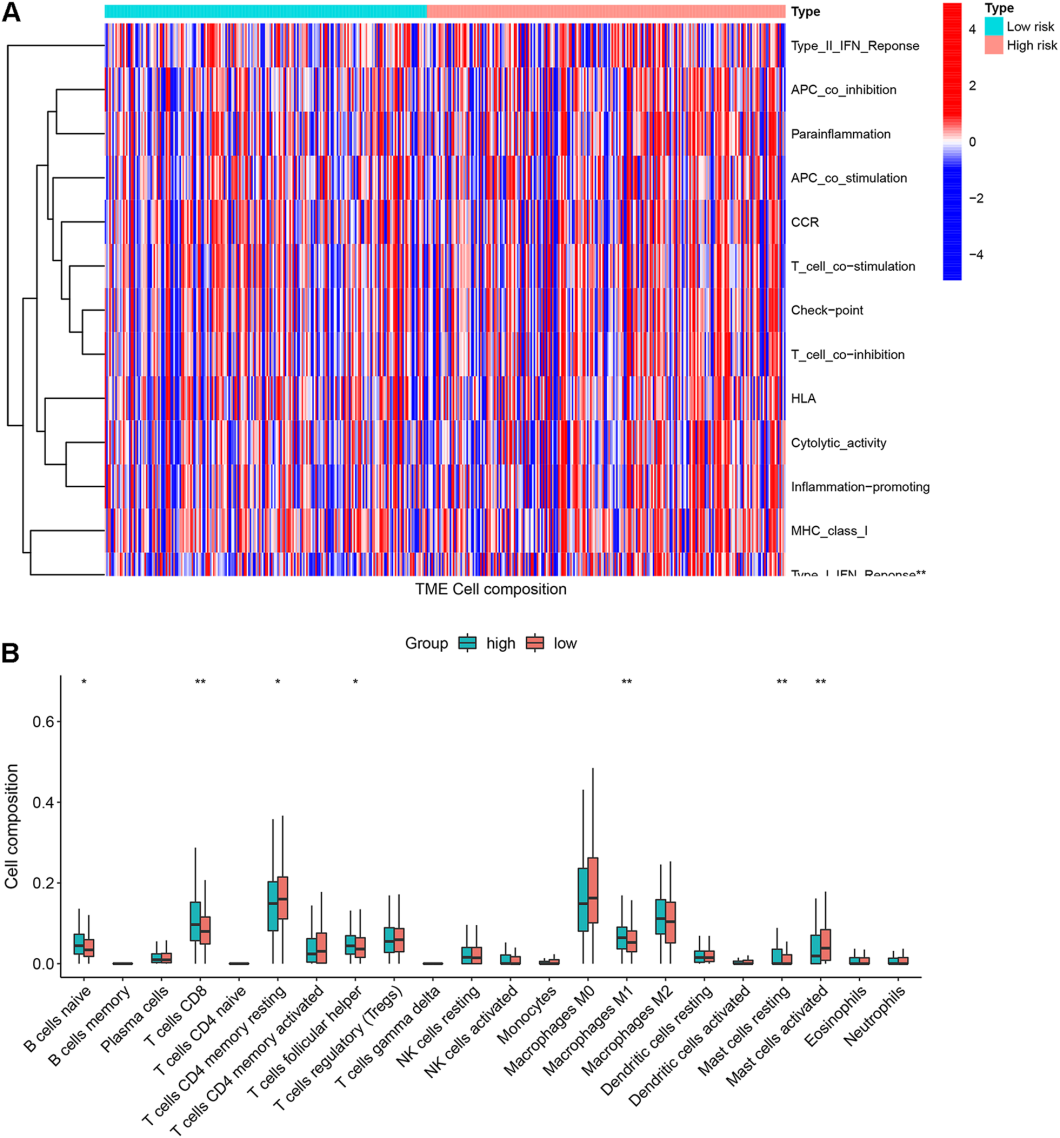
Heatmap of the relationship between cuproptosis-related genes and the immune characteristics of CRC between the low- and high-risk groups (**A**). Boxplots of the abundance of the 22 immune cells between the high-risk and low-risk groups, *p < 0.05, **p < 0.01 (**B**)

We applied the CIBERSORT algorithm to estimate the abundance of TIICs to explore the correlation between the risk score and TIIC characteristics. The results revealed that the abundance of TIICs in the low-risk groups, including naïve B cells, CD8^+^ T cells, follicular helper T cells, M1 macrophages and resting mast cells, was significantly increased compared with that in the high-risk groups. However, the abundance of CD4^+^ T memory resting cells and mast cells activated in the high-risk groups was greater than that in the low-risk groups (Fig. 11).

### Gene mutations in the high-risk and low-risk groups

**Fig. 12:**
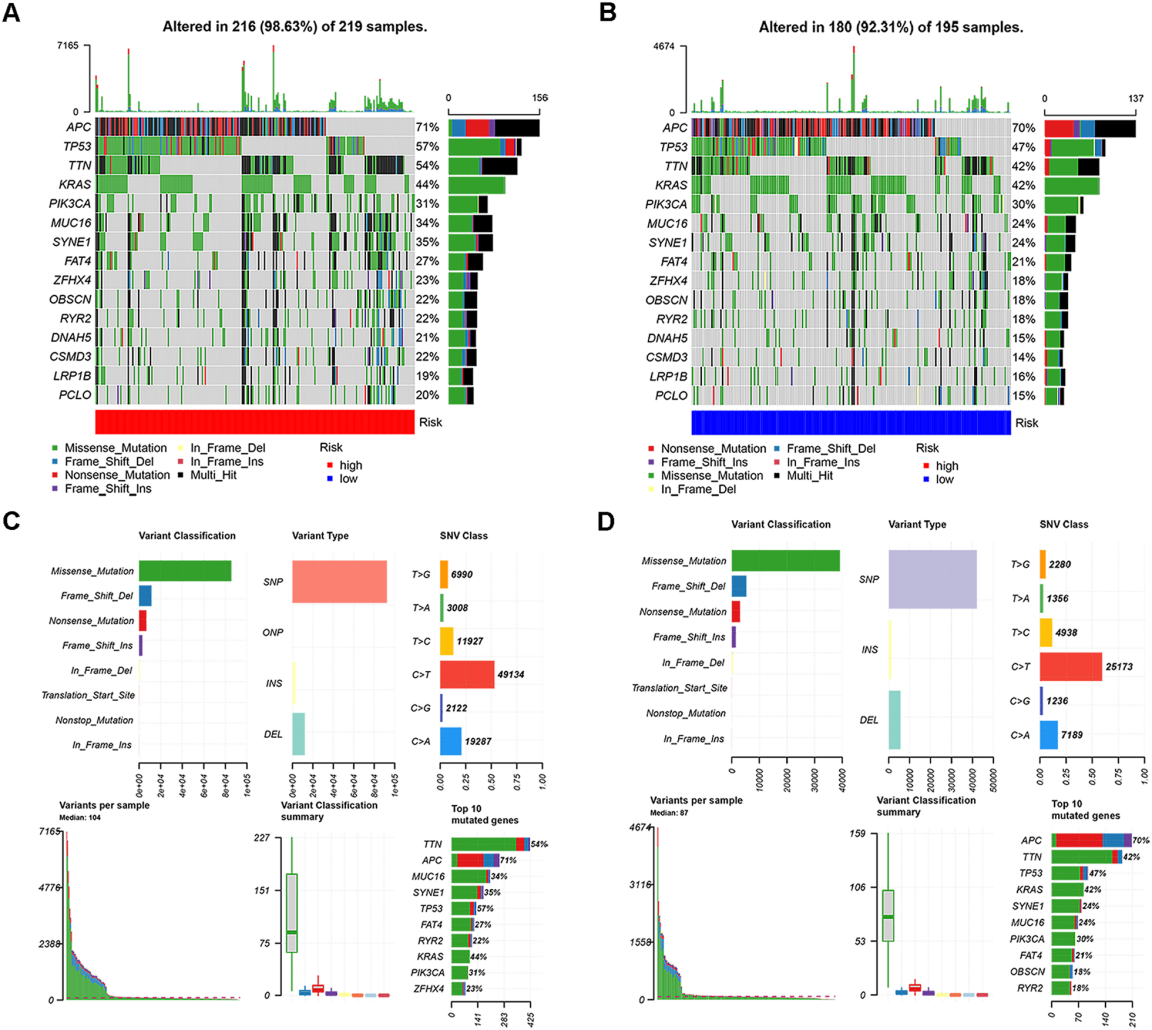
MAF-summary plots and oncoplots of the somatic mutation between the high-risk (**A**, **C**) and low-risk (**B**, **D**) groups in the TCGA dataset

**Fig. 13:**
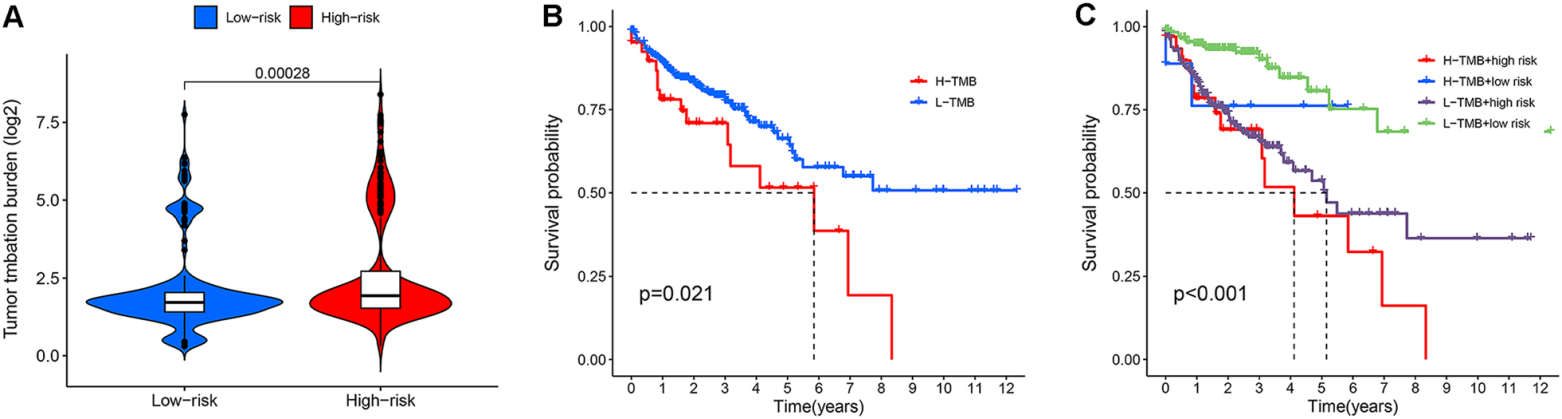
Violin plot for the TMB scores between the high-risk and low-risk groups (**A**). Kaplan–Meier survival curve of the risk score in the high-mutation and low-mutation groups (**B**). Kaplan–Meier survival curve of the risk score in the high-mutation + high-risk, high-mutation + low-risk, low-mutation + high-risk, and low-mutation + low-risk groups (**C**)

We downloaded mutation profiles (MAF) of CRC patients from TCGA datasets and visualized 15 genes with the highest mutation levels. Then, we generated waterfall plots based on the mutation frequency of the high-risk and low-risk groups. The results revealed that the mutation frequency of 15 genes in the high-risk group was greater than that in the low-risk group (Fig. 12A, B), and the main gene information is presented in the bar plot (Fig. 12C, D). The top 5 mutated genes in the high-risk group were APC (71%), TP53 (57%), TTN (54%), KRAS (44%) and PIK3CA (31%), and the top 5 mutated genes in the low-risk group were APC (70%), TP53 (47%), TTN (42%), KRAS (42%) and PIK3CA (30%). TP53, MUC16 and SYNE1 expression was increased in the high-risk group compared with the low-risk group. In addition, we further analysed the TMB of the high-risk and low-risk groups. The levels of gene mutation in the high-risk group were observably increased compared with those in the low-risk group (Fig. 13A). To evaluate the predictive value of the risk score for the prediction of tumour burden survival, we divided CRC patients into high-mutation and low-mutation groups based on the median TMB and then plotted survival curves of the risk score in the mutation subgroups. As shown in Fig. 13B, C, CRC patients in the low-mutation group had better survival outcomes, and the survival outcomes of 4 groups, including the high-risk + high-mutation group, high-risk + low-mutation group, low-risk + high-mutation group and low-risk + low-mutation group, were also significantly different.

### Drug sensitivity analysis

**Fig. 14:**
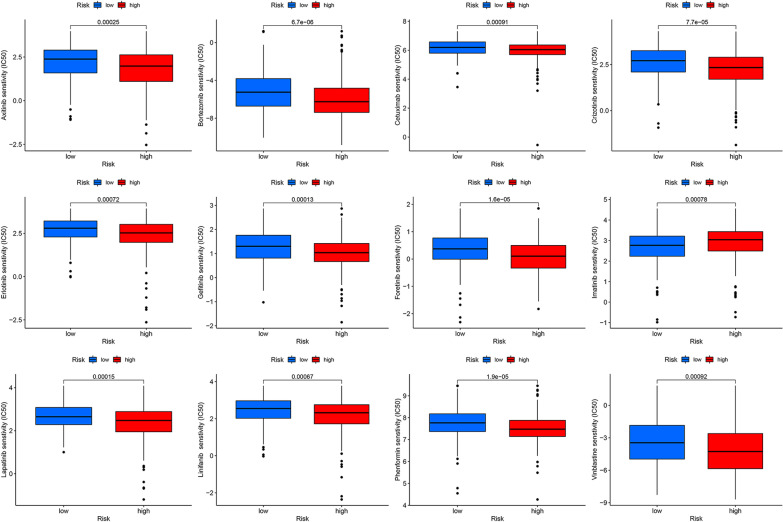
Box plot showing the mean differences in estimated IC50 values of 12 representative drugs between the two risk groups

In addition, we further analysed the relationship between the risk score and drug sensitivity. Then, we used box plots to compare chemotherapeutic effects or the estimated half inhibitory concentration (IC50) in the high-risk and low-risk groups. The results indicated that 11 drugs (axitinib, bortezomib, cetuximab, crizotinib, erlotinib, foretinib, gefitinib, lapatinib, linifanib, phenformin and vinblastine) may be beneficial for the treatment of patients in the high-risk group, whereas imatinib may be harmful for patients in the high-risk group (Fig. 14).

### The expression of SNHG16, LINC02257, RPARP-AS1, LENG8-AS1 in CRC

**Fig. 15:**
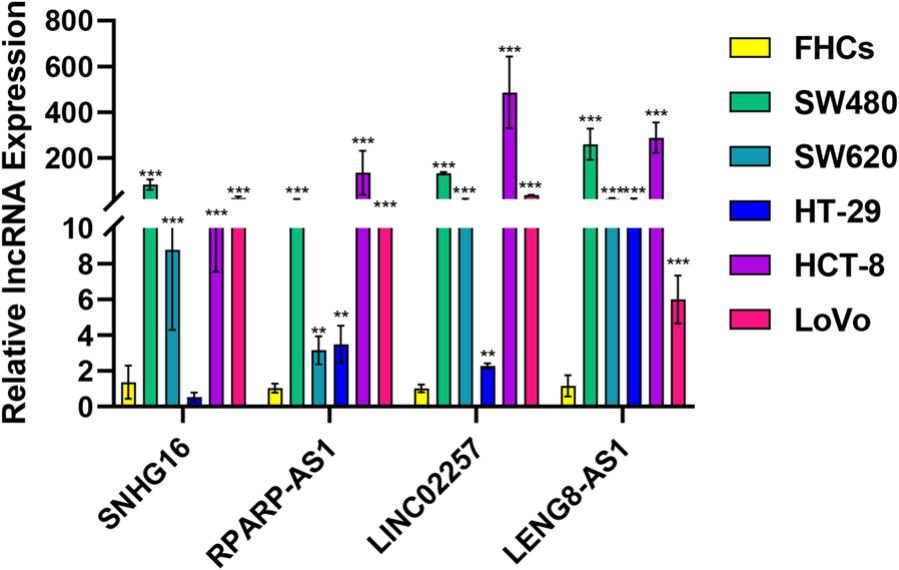
The expression of SNHG16, LINC02257, RPARP-AS1, LENG8-AS1 in CRC cell lines

To further explore the expressions of SNHG16, LINC02257, RPARP-AS1, LENG8-AS1. Human intestinal epithelial cells (FHCs) and human colorectal cancer cell lines (SW480, SW620, HCT8, HT29, LoVo) were used to validate the expression levels of the four lncRNAs. Quantitative real-time PCR (qRT-PCR) analysis results performed that SNHG16, LINC02257, RPARP-AS1, LENG8-AS1 were differentially expressed in CRC cell lines compared to that in intestinal epithelial normal cell lines (Fig. 15). Moreover, these results showed that SNHG16, LINC02257, RPARP-AS1, LENG8-AS1 may play an important role in CRC.

## Discussion

Previous studies have focused on the function of lncRNAs in ferroptosis. The recent discovery of cuproptosis-related genes has attracted considerable attention [28–30]. Copper-induced cell death is caused by direct binding to the lipoylated components of the tricarboxylic acid (TCA) cycle. These effects result in the aggregation of lipoylated proteins, loss of proteins containing Fe-S clusters, and induction of HSP70, leading to proteotoxic stress and ultimately cell death [14]. Feng et al. [31] constructed the cuproptosis-related lncRNA (CRLs) nomogram model by Cox regression analysis with the Lasso algorithm to predict the prognosis of gastric cancer patients. Wang et al. [32] found the involvement of cuproptosis-related genes regulation in Hepatocellular carcinoma (HCC). Li et al. [33] develop the cuproptosis-related prognostic signature to predict the prognosis of breast cancer (BC) through the Cox and Lasso regression analyses. However, studies of the function of CRLs in colorectal cancer (CRC) remain limited. This present study identified CRLs (SNHG16, LINC02257, RPARP-AS1, and LENG8-AS1) in CRC and their prognostic value that provides a foundation and new latent therapeutic targets for prognosis and clinical treatment.

Many studies have revealed that SNHG16 plays a vital role as an oncogene in numerous cancers [34–39] through various of pathways. SNHG16 sponged miR-135a and promoted Janus-activated kinase 2 (JAK2) and transcription Factor 3 (STAT3) expression in gastric cancer [38]. SNHG16 regulates the target ZEB1 by competing with miR-140-5p as an endogenous "sponge" that promotes oesophageal squamous cell carcinoma [40]. A study found that SNHG16 promotes CRC cell proliferation, migration, and EMT through the miR-124-3p/MCP-1 axis [11]. In addition, SNHG16 is regulated by the Wnt pathway and is involved in lipid metabolism in clinical tumours. As a ceRNA, SNHG16 competes with miRNA for the 3'UTR of stearoyl‐CoA desaturase, which "sponges" miRNAs off their cognate targets [22]. In our study, we found that SNHG16 were significantly correlated with adverse survival in CRC. The cuproptosis-related genes may play an essential role in CRC development, proliferation, and migration.

Currently, most studies determine that LINC02257 can serve as a prognostic marker. Gauteng Lin et al. used weighted co-expression network analysis to select lncRNAs, including LINC02257 and LINC01820, to construct a renal clear cell carcinoma (KIRC) model and analysed the potential value of LINC02257 in prognosis [41]. Based on differential lncRNA expression between CRC patients and normal groups, Xiao Huang et al. constructed a LASSO model to screen six lncRNAs, containing LINC02257, and developed a CRC survival prediction model [42]. Wang et al. used the optimal cut-off value, which was applied to the Youden index, based on the differential expression between different tissues to divide the patients into high-risk and low-risk groups and constructed a survival prediction model using 15 lncRNAs [13]. In addition, Xiao et al. [43] applied multiomics methods to demonstrate LINC02257 expression, which is associated with multiple poor outcomes in various cancers and serves as an independent prognostic biomarker for colon adenocarcinoma through the PI3K-Akt signalling pathway. All of the above studies suggest that LINC02257 is of great value in CRC survival prediction. Our study showed that LINC02257 is associated with adverse outcomes in CRC, which provided evidence that LINC02257 can potentially affect the prognosis of CRC patients.

Bu et al. [44] identified RPARP-AS1 as a pyroptosis-related lncRNA and investigated its prognostic value in osteosarcoma. Li et al. [45] found that RPARP-AS1 could serve as a biomarker associated with breast cancer prognosis. Ren et al. [46] experimentally demonstrated that RPARP-AS1 acts as a competitive endogenous RNA (ceRNA) to sponge miR-125a-5p, thus promoting CRC proliferation, migration, and invasion.

In contrast to SNHG16 and PRARP-AS1, only the prognostic value of LINCO2257 has been demonstrated, and experiments are required to confirm how it regulates the tumour proliferation and migration. There are no reports on the prognostic value of LENG8-AS1 in cancer. Further studies are still needed to assess the role of LENG8-AS1 as a related prognostic gene or as a novel therapeutic target.

This study identified four markers of CRC prognosis using univariate and multivariable Cox regression: SNGH16, LINC02257, PRARP-AS1, and LENG8-AS1. Then, we constructed risk scores based on these four prognostic markers that were good predictors of CRC prognosis and could be independent risk features for CRC prognosis. Next, we divided CRC patients into high-risk and low-risk groups according to the risk score, further exploring high-risk and low-risk clinical characteristics. We found that the risk score could also predict other adverse prognoses of CRC patients. Furthermore, to explore how cuproptosis-related lncRNAs regulate the development of CRC in high-risk and low-risk groups, we performed KEGG and GO enrichment for differential CRLs in high-risk and low-risk groups. These findings demonstrated that differential genes were involved in the "PPAR" signalling pathway. Some studies have shown that the “PPAR” signalling pathway is overexpressed in cancers, such as gastric cancer [47], cervical cancer [48], and oesophageal cancer [49]. Other studies have also shown that PPAR has antagonistic effects on lung, breast, prostate, and colon cancers [50]. Thus, PPAR may provide a new direction for the treatment of CRC. Ferroptosis is mainly characterized by the depletion of glutathione and decreased activity of glutathione peroxidase 4 (GPX 4); lipid oxides cannot be reduced, producing large amounts of reactive oxygen species, leading to cell death [51]. The enrichment analysis showed that the differences between the high-risk and low-risk groups reflected redox characteristics, such as sulfur compound binding, metabolism of xenobiotics by cytochrome P450, lysosomal and lipid pathways. Therefore, we infer some biological associations between cuproptosis and ferroptosis. It is expected to be a novel therapeutic target. Finally, we also found that the differentially expressed genes in the high-risk and low-risk groups were also involved in cancerous pathways, such as chemical carcinogenesis-DNA adducts and basal cell carcinoma which may provide a new direction for the treatment of CRC. Moreover, we found that the expression of SNHG16, LINC02257, RPARP-AS1, LENG8-AS1 were differentially expressed in CRC cell lines compared to that in intestinal epithelial normal cell lines.

In this study, we analysed the proportion of tumour-infiltrating immune cells, including naïve B cells, CD8 + T cells, follicular helper T cells, M1 macrophages, and resting mast cells, between the high-risk and low-risk groups using the CIBERSORT algorithm. We found that the abundance of TIICs in the low-risk group was significantly greater than that in the high-risk group. Previous studies have shown that CD4 + and CD8 + T-cell responses are part of the cancer-immune cycle and that both parts can significantly influence the clinical outcome [52]. Furthermore, our study showed that the abundance of TIICs, such as T cells, resting memory CD4 T cells, and activated mast cells, was lower in the low-risk group compared with the high-risk group. These results are consistent with the results of Yang et al. [53]. Besides, our study showed that the abundance of TIICs, such as resting memory CD4 T cells and activated mast cells, was lower in the low-risk score group compared with the high-risk group. Moreover, the environment of tumour-infiltrating immune cells represents the immune status of CRC patients, which may explain the differences between high-risk and low-risk patients.

We also found a correlation between prognostic CRLs and TMB in the high-risk and low-risk groups, and APC, TP53, and KRAS all showed high expression in the high-risk and low-risk groups. P H Cottu et al. also revealed that APC, TP53, and KRAS were related to cancer occurrence and progression [54]. We found that TP53, MUC16, and SYNE1 expression was significantly increased in the high-risk group compared with the low-risk group. Numerous studies have shown that TP53, a tumour suppressor gene, is one of the crucial elements of human defence against cancer [55]. Previous studies have also shown that MUC16 functions in tumour proliferation, metastasis, and inhibition of natural killer cells to regulate the innate immune response [56, 57]. Nevertheless, no relevant reports have revealed that SYNE1 is related to the occurrence of CRC. We also found significant prognostic differences between somatic mutations in the high-risk and low-risk groups. This finding indicated that a possible relationship might exist between prognostic CRL genes and mutations in TPS, MUC16, and SYNE1. However, this relationship should be investigated further.

There are some limitations to this study. First, the prognostic value of CRLs in CRC was verified exclusively using external data because external RNA-seq data are challenging to obtain and lack clinical features. In the next steps, these findings need to be further confirmed by continuously expanding the research data. Second, this study only used four CRLs that showed good predictive value and did not explore the other relationships between cuproptosis and related lncRNAs in depth. We need to explore the exact relationship between the two factors. Third, there are some over-fitting situations in TCGA testing set. Finally, despite the essential prognostic value of cuproptosis-related lncRNA signature identified in this study, future experiments on lncRNAs components are required to elucidate their roles in CRC.

## Conclusions

In conclusion, this study identified the cuprotosis-related lncRNAs (CRLs) in CRC, established a prognostic risk model that includes 4-CRLs, and explored the potential prognostic value of CRLs in CRC. The findings of this study may provide new insights into CRC prognosis studies and contribute to the development of clinical treatments for CRC.

## Supplementary Information

**Additional file 1: Table S1. **The primers sequences of four cuproptosis-related lncRNAs.

**Additional file 2: Fig. S1. **Flow chart for this study. **Fig. S2.** The area under the ROC curve of models of 18 CRLs. **Fig. S3.** The comparison of 4-CRLs score model and 18-CRLs model. **Fig. S4. **The area under the ROC curve incorporating the Nomogram model and clinical characteristics to predict the 1-(A), 3- (B) and 5-year(C) overall survival rates of patients with CRC on TCGA training set.

**Additional file 3: Table S2**. Clinical features of the patients with CRC in each cohort.

## Data Availability

The data of this study were obtained from the publicly available database. RNA-sequencing profile data and corresponding clinical data for colon cancer were extracted from TCGA (https://tcga-data.nci.nih.gov/tcga/). Previously reported cuproptosis-related genes data were used to support this study and are available at [https://doi.org/10.1126/science.abf0529.] Infiltration data for 22 immune cells were downloaded from the TIMER (http://timer.cistrome.org) and CIBERSORT (https://cibersort.stanford.edu/) databases. Data used in the paper could be derived in a public repository to share. The original contributions presented in the study are included in the article/Supplementary Materials, The original contributions presented in the study are included in the article/supplementary materials, further inquiries can be directed to the corresponding author.
