# Supplementary material for: Development and validation of cuproptosis-related lncRNA signatures for prognosis prediction in colorectal cancer
Source: BMC Med Genomics. 2023 Mar 22;16:58. doi: 10.1186/s12920-023-01487-x (PMC10031908; doi:10.1186/s12920-023-01487-x)
Supplement: Supplementary file 2 — Additional file 2: Fig. S1. Flow chart for this study. Fig. S2. The area under the ROC curve of models of 18 CRLs. Fig. S3. The comparison of 4-CRLs score model and 18-CRLs model. Fig. S4. The area under the ROC curve incorporating the Nomogram model and clinical characteristics to predict the 1-(A), 3- (B) and 5-year(C) overall survival rates of patients with CRC on TCGA training set. [file 12920_2023_1487_MOESM2_ESM.docx]

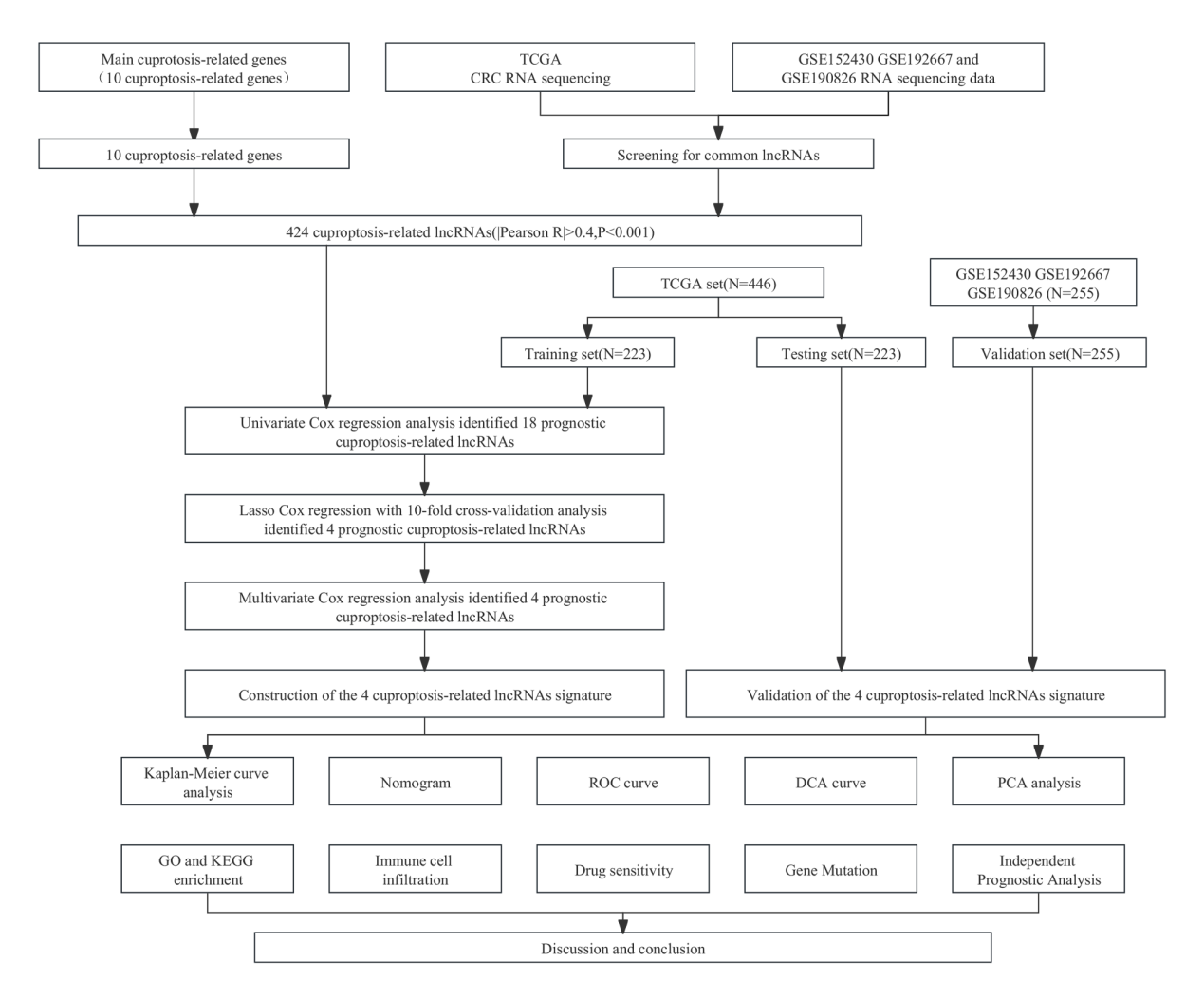


**Fig. S1** Flow chart for this study.


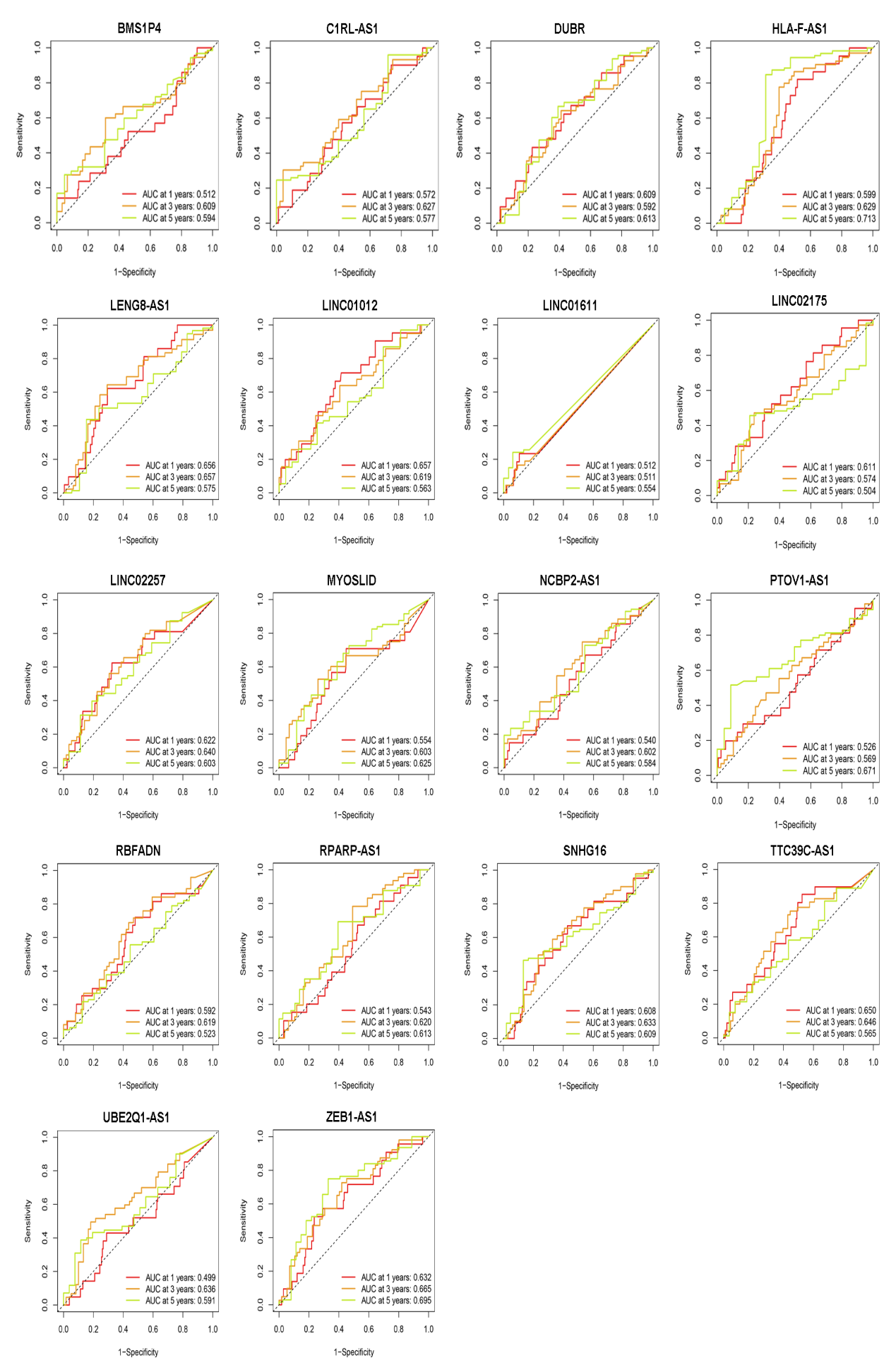


**Fig. S2** The area under the ROC curve of models of 18 CRLs.


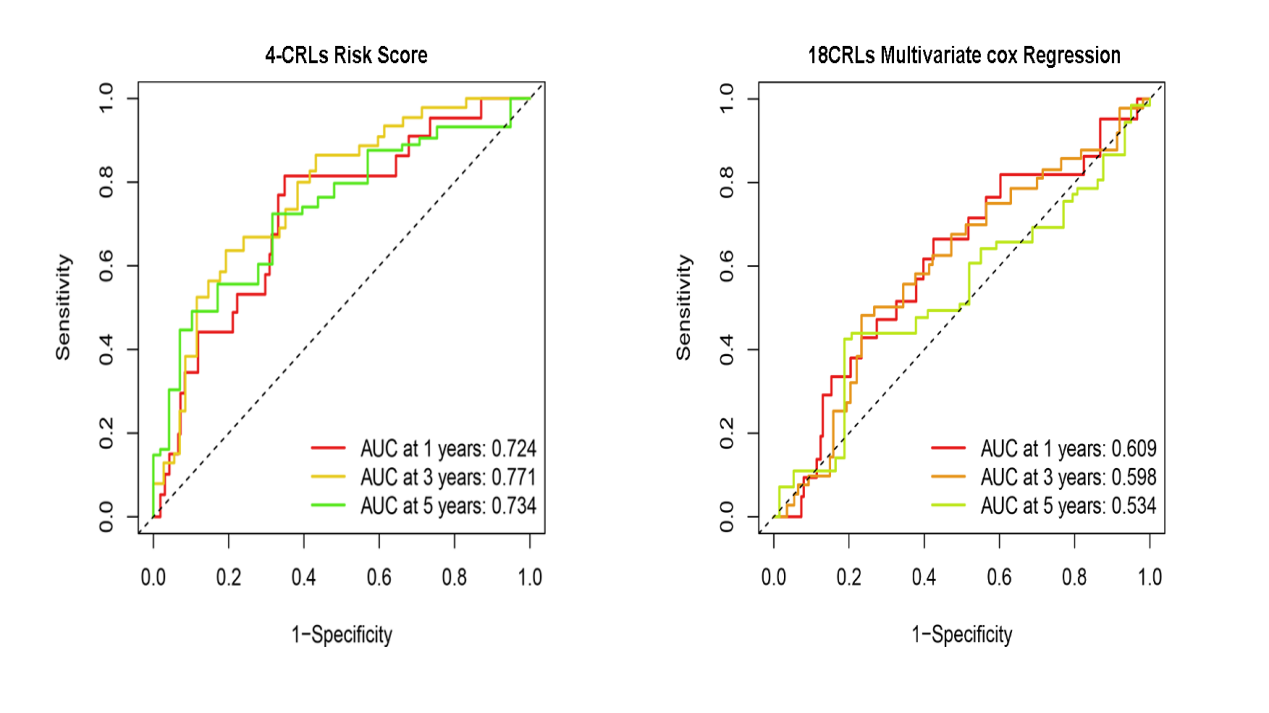


**Fig. S3** The comparison of 4-CRLs score model and 18-CRLs model.


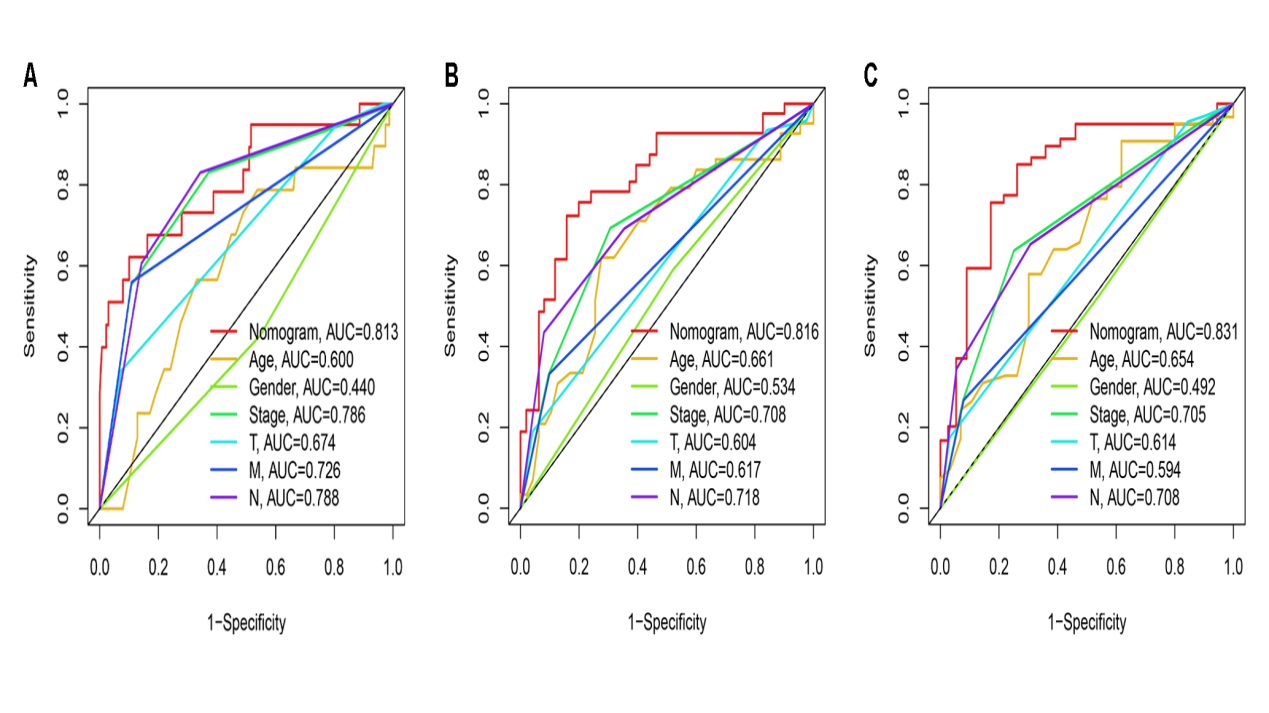


**Fig. S4** The area under the ROC curve incorporating the Nomogram model and clinical characteristics to predict the 1-(A), 3- (B) and 5-year(C) overall survival rates of patients with CRC on TCGA training set.
