# Supplementary material for: Development and validation of cuproptosis-related lncRNA signatures for prognosis prediction in colorectal cancer
Source: BMC Med Genomics. 2023 Mar 22;16:58. doi: 10.1186/s12920-023-01487-x (PMC10031908; doi:10.1186/s12920-023-01487-x)
Supplement: Supplementary file 3 — Additional file 3: Table S2. Clinical features of the patients with CRC in each cohort. [file 12920_2023_1487_MOESM3_ESM.docx]

**TABLE S2|** Clinical features of the patients with CRC in each cohort

| Variables | Training  cohort (n = 223) | Testing  cohort (n = 223) | *P* |
| --- | --- | --- | --- |
| Survival status |  |  | 0.249 |
| Alive | 170(76.2.0%) | 180(80.7%) |  |
| Dead | 53(23.8%) | 43(19.3%) |  |
| Age(years) |  |  | 0.630 |
| ≤65 | 94(42.2%) | 89(39.9%) |  |
| ＞65 | 129(57.8%) | 134(60.1%) |  |
| Gender |  |  | 0.255 |
| Female | 112(50.2%) | 100(44.8%) |  |
| Male | 111(49.8%) | 123(55.2%) |  |
| Tumor invasion (T) |  |  | 0.264 |
| T1 | 4(1.8%) | 6(2.7%) |  |
| T2 | 40(17.9%) | 36(16.1%) |  |
| T3 | 157(70.4%) | 146(65.5%) |  |
| T4 | 22(9.9%) | 35(15.7%) |  |
| Lymph node (N) |  |  | 0.391 |
| N0 | 137(61.4%) | 128(57.4%) |  |
| N1 | 52(23.3%) | 50(22.4%) |  |
| N2 | 34(15.2%) | 45(20.2%) |  |
| Metastasis (M) |  |  | 0.290 |
| M0 | 165(74.0%) | 164(73.5%) |  |
| M1 | 26(11.7%) | 35(15.7%) |  |
| Unknown | 32(14.3%) | 24(10.8%) |  |
| Tumor stage |  |  | 0.631 |
| Stage I | 39(17.5%) | 36(16.1%) |  |
| Stage II | 91(40.8%) | 84(37.7%) |  |
| Stage III | 63(28.3%) | 61(27.4%) |  |
| Stage IV | 26(11.7%) | 35(15.7%) |  |
| Unknown | 4(1.8%) | 7(3.1%) |  |
